# Challenges of Constipation in People Suffering from Schizophrenia: A Narrative Review

**DOI:** 10.3390/clinpract15020033

**Published:** 2025-02-11

**Authors:** Takahiko Nagamine

**Affiliations:** 1 Department of Psychiatric Internal Medicine, Sunlight Brain Research Center, Hofu 7470066, Japan; tnagamine@outlook.com; Tel.: +81-3-3726-1111; 2 Department of Psychosomatic Dentistry, Graduate School of Medical and Dental Sciences, Institute of Science Tokyo, Bunkyou 1138510, Japan

**Keywords:** schizophrenia, constipation, clozapine, sex differences, descending pain inhibitory pathway, Ogilvie’s syndrome, laxatives, probiotics

## Abstract

**Background/Objectives**: Individuals diagnosed with schizophrenia are susceptible to constipation induced by antipsychotic medications. However, research investigating the prevalence of this adverse effect and its underlying mechanisms is limited. **Methods**: To address this knowledge gap, a narrative review was conducted on the subject of constipation in patients with schizophrenia. A comprehensive electronic search was conducted in the PubMed and J-Stage databases. **Results**: The prevalence of constipation in individuals with schizophrenia is high, ranging from 30% to 60%. The risk of constipation increases with high doses of antipsychotics and with the concomitant use of anticholinergics and mood stabilizers. The prevalence of constipation varies by sex, with women demonstrating a higher risk compared to men. **Conclusions**: Constipation is a prevalent complaint among individuals with schizophrenia, with women exhibiting a higher risk. The underlying pathophysiology of sex differences in constipation is attributed to variations in monoamines within the descending pain inhibitory pathway, which governs the neural circuitry associated with defecation. Constipation can lead to significant complications, underscoring the need for a multifaceted approach to treatment that considers the intestinal environment beyond the mere use of laxatives.

## 1. Introduction

Antipsychotic medications are the cornerstone of the management for individuals with schizophrenia. However, the quality of life (QOL) of people with schizophrenia taking antipsychotics remains very poor, and this outcome is rarely considered in trials evaluating the efficacy and effectiveness of antipsychotic treatment [1]. Antipsychotic medications represent the fundamental treatment approach for schizophrenia; however, they are associated with numerous adverse effects. Among these, constipation is a prevalent concern and can present as a serious adverse effect [2]. Constipation is a prevalent adverse effect among individuals diagnosed with schizophrenia, yet its prevalence and impact in clinical trials remain under-explored. The severity of constipation can range from mild to severe, and its consequences can be serious, including but not limited to paralytic ileus, intestinal obstruction, and death [3]. There is currently no active screening or monitoring for constipation in clinical practice, and as a result, the incidence, prevalence, and underlying mechanisms are often not known [4]. The definition of constipation is subjective and multifaceted, involving numerous factors such as the frequency of bowel movements, stool quality, and the sensation of incomplete evacuation. Constipation is influenced by a multitude of factors, including lifestyle, intestinal flora, and stress. Consequently, there is a paucity of systematic studies on constipation in individuals with schizophrenia [5]. This manuscript is a narrative review that seeks to identify objective data on constipation in patients with schizophrenia in clinical settings and to discuss its associated problems and mechanisms.

## 2. Materials and Methods

**Figure 1:**
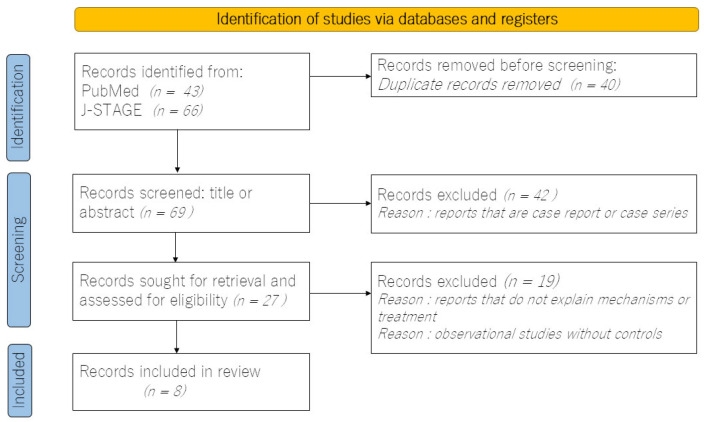
Flow diagram for the review, which included searches of databases and registers.

A comprehensive electronic search was conducted on the PubMed and J-stage databases for articles in English that contained the keywords “schizophrenia”, “constipation”, “antipsychotics”, and “complications” in the title or abstract. The search covered the period from January 2015 to December 2024. The full text of the retrieved articles was examined, excluding case series. Subsequently, studies that did not address mechanisms or treatments, or that lacked a comparison control group, were excluded. The initial screening and selection process was conducted by the author. Initially, 69 abstracts were reviewed; however, the majority were case series reports, and there were no randomized controlled trials, resulting in a limited number of studies that met the study’s objectives. Following this thorough review, a total of eight observational studies were included in the final review. However, the majority of these studies were secondary analyses of registered datasets rather than studies specifically focusing on constipation. The flowchart illustrating the search process is presented in Figure 1.

## 3. Results

None of the papers included in this review employed the strict Rome IV criteria for defining constipation in individuals with psychiatric illness. Rather, they defined constipation based on subjective assessment or the use of laxatives. However, we were able to identify observational studies that mentioned discomfort, complications, and the treatment of constipation in individuals with schizophrenia. The majority of these reports have indicated an escalating trend in the prevalence of constipation among individuals diagnosed with schizophrenia.

A study was conducted to examine the concomitant use of laxatives in older individuals with schizophrenia who were discharged from the hospital after receiving atypical antipsychotics. The study found that the rate of laxative use increased over time, from 30.8% in 2006 to 46.6% in 2019 [6]. Multivariate logistic regression analysis identified female sex, the use of mood stabilizers, and comorbid diabetes as risk factors for laxative use. Among the various antipsychotic medications, clozapine was associated with the highest risk, followed by zotepine, quetiapine, and olanzapine [6]. In a cross-sectional study of 271 participants aged 20–65 years, the prevalence of constipation was 66.4%, and multivariate logistic regression analysis identified risk factors for constipation as female gender, anticholinergic drugs, depression, and anxiety symptoms [7]. In a separate study focusing on atypical antipsychotics, 11,861 patients treated with these medications and discharged from the hospital were identified. Of these patients, 3336 (28.1%) were prescribed laxatives concomitantly. Multivariate analysis revealed that factors associated with laxative prescription included older age, high doses of antipsychotics and anticholinergics, and the prescription of clozapine. Furthermore, regular laxative use at discharge was significantly associated with psychiatric rehospitalization [8].

As previously indicated, one of the risk factors for constipation in patients diagnosed with schizophrenia is being female, and there are gender disparities in the prevalence of constipation. While the efficacy of clozapine remained consistent across both sexes, biological sex disparities emerged concerning the manifestation of adverse effects. Specifically, women exhibited a higher propensity for constipation, weight gain, and the onset of diabetes, in contrast to men [9]. A secondary analysis of data from the Clinical Antipsychotic Trials of Intervention Effectiveness (CATIE) study examining sex differences in the efficacy and side effects of antipsychotics found no differences between men and women in antipsychotic dosage, medication adherence, response to antipsychotics, or extrapyramidal symptoms. However, constipation (28% vs. 16%), dry mouth (50% vs. 38%), gynecomastia/galactorrhea (11% vs. 3%), incontinence/nocturia (16% vs. 8%), and weight gain (37% vs. 24%) were significantly more prevalent in female participants than in male participants (all *p* < 0.001) [10].

As previously mentioned in the section on risk factors, clozapine, an antipsychotic-associated drug, was identified as the most significant contributor to constipation. Moreover, clozapine was associated with an increased prevalence of severe constipation complications. In a survey on laxative use, a study of 93 individuals with schizophrenia in a psychiatric rehabilitation hospital used multivariate analysis to examine changes in laxative dosage over a one-week period and found that clozapine dosage, age, diabetes, and hypothyroidism were risk factors for increased laxative dosage [11]. In a subsequent study on clozapine prescriptions and laxatives, among 53 hospitalized individuals with schizophrenia who were switched to clozapine due to treatment resistance, magnesium oxide and lubiprostone were prescribed more frequently with clozapine. Furthermore, older age, a longer duration of illness, and insulin resistance were associated with the use of these laxatives [12]. The incidence of serious complications from constipation was examined in a group of 188 constipated individuals treated with clozapine. The study found that four of these individuals developed ileus, two of whom required a permanent stoma due to the ileus [13].

## 4. Discussion

### 4.1. Mechanism of Antipsychotic-Induced Constipation

The prevalence of constipation is high in patients diagnosed with schizophrenia, ranging from 30% to 60%. Atypical antipsychotics constitute the primary treatment modality, yet the prevalence of constipation is increasing [5]. The multifactorial nature of constipation in these patients, involving both psychological and physiological elements, has been extensively researched. Among the psychological factors, the decline in gastrointestinal motility induced by psychotropic drugs has been identified as a significant contributing factor. Risk factors for constipation that have been identified in various studies include female gender, high doses of antipsychotics, concomitant use of mood stabilizers, concomitant use of anticholinergic drugs, and metabolic disorders [9].

Atypical antipsychotics act on various receptors other than dopamine D2 receptors. Clozapine, quetiapine, and olanzapine have been associated with constipation [10], and all of these medications possess a tricyclic structure and exhibit robust anticholinergic properties, which can potentially lead to the suppression of gastrointestinal motility. These medications also exhibit significant interactions with muscarinic receptors, histamine H2 receptors, and serotonin 5HT2C receptors, which can potentially lead to metabolic disturbances [14]. Constipation has been identified as a risk factor for diabetes mellitus, and constipation may be exacerbated by metabolic disorders [5]. Furthermore, the concomitant administration of antipsychotics and anticholinergics or mood stabilizers has been associated with an increased risk of bowel problems due to the blockade of neurotransmitters at multiple receptors. This increased risk may be attributable to the occurrence of gastrointestinal motility disorders and metabolic disorders, which are potentially mediated by various receptors, such as those affected by clozapine administration.

### 4.2. The Role of Dopamine in the Defecation Reflex

The relationship between the D2 receptor blockade, the primary pharmacological action of antipsychotics, and the defecation reflex has not been thoroughly examined until now. However, the potential involvement of dopamine in both central and peripheral components of the defecation reflex is a subject that merits further investigation. The defecation reflex is initiated when nociceptive stimulation of the colonic extension is transmitted to the brain by primary afferent neurons via ascending pain pathways. The brain’s response to nociceptive colonic distension involves the stimulation of the periaqueductal gray (PAG) via dopaminergic neurons. Consequently, the descending pain inhibitory pathways originating from the PAG are activated, leading to the alleviation of pain perception and the induction of the defecation reflex. The process of descending pain inhibitory pathway activation involves the release of neurotransmitters such as serotonin, noradrenaline, dopamine, and gamma-aminobutyric acid (GABA) into the spinal cord. These monoamines regulate the activity of the sacral nerves. The defecation reflex is initiated when the descending pain inhibitory pathway originating from the PAG stimulates the sacral spinal cord’s defecation center [15]. Antipsychotics have been observed to block dopamine neurons, thereby reducing PAG activation and potentially impacting the integrity of the defecation reflex.

In addition, recent animal models have demonstrated the significance of monoamines in the defecation reflex in disease models. A notable example is Parkinson’s disease (PD), where patients often experience constipation, and the underlying mechanisms have been investigated in animal models. These studies have demonstrated a significant role of dopamine D2 receptors in the regulation of the defecation reflex. The creation of PD model rats involved the injection of 6-hydroxydopamine into the unilateral medial forebrain bundle, leading to the destruction of dopaminergic neurons in the substantia nigra. Colonic motility is induced by the administration of capsaicin, a noxious stimulus, into the intestine. Intra-abdominal administration of capsaicin has been observed to induce colonic motility in rats subjected to sham operations, yet this response is absent in rats modeling PD. Intraspinal administration of dopamine and serotonin to PD model rats induced colonic motility, and this capsaicin-induced colonic motility was abolished by the intraspinal administration of a D2 receptor antagonist [16]. Furthermore, experiments on the defecation reflex in PD model rats have demonstrated that the absence of central D2 receptor functionality can result in the suppression of both the afferent and efferent pathways involved in defecation.

In the context of Parkinson’s disease (PD) modeling, rats treated with high doses of antipsychotics have been shown to exhibit characteristics that are analogous to those observed in PD patients. Specifically, the administration of antipsychotics has been demonstrated to attenuate both the afferent and efferent pathways involved in the defecation reflex. Constipation is associated with the impairment of the central nervous system’s regulation of colonic motility, and since dopamine D2 receptors regulate this system, antipsychotics, dose-dependently, reduce the defecation reflex, thereby posing a risk of constipation.

### 4.3. Mechanism of Sex Differences in the Defecation Reflex

The prevalence of constipation exhibited sex-based disparities, with women demonstrating a heightened risk [7]. Data concerning the efficacy and tolerability of antipsychotics in schizophrenia patients show no sex differences in efficacy; however, in terms of tolerability, women are more likely to experience side effects, and the prevalence of constipation is higher in women than in men [8]. It is imperative to consider sex-specific differences in drug metabolism as a potential explanation for the heightened sensitivity of female patients to adverse effects. While variations in the concentration of antipsychotics are attributable to cytochrome P450 and p-glycoprotein, the impact of these enzyme reactions is predominantly influenced by genetic polymorphisms, which are less likely to account for observed sex differences. The observed discrepancy in the prevalence of constipation between sexes cannot be fully attributed to these enzyme polymorphisms.

The propensity of female subjects to report constipation may be attributable to sex-related disparities in the circuit of the defecation reflex. The efferent pathway of the defecation reflex is initiated by the activation of the descending pain inhibitory pathway, which originates from the PAG. Animal models have demonstrated clear gender disparities in the expression of neurotransmitters within the descending pain inhibitory pathway. In male subjects, the release of dopamine and serotonin activates the defecation center in the sacral spinal cord, thereby promoting colonic motility. In contrast, in women, the release of serotonin and GABA occurs, with serotonin promoting defecation and GABA inhibiting it, resulting in a lower defecation reflex compared to men [17]. The prevalence of defecation not only varies by gender, but also by age, with a higher incidence observed in both schizophrenia and in the female population. The decline in the defecation reflex in women may be influenced by variations in the composition of monoamines within the descending pain inhibitory system.

### 4.4. Ogilvie’s Syndrome as a Severe Complication of Constipation

Constipation is a multifaceted condition that impacts both the physical and mental aspects of an individual’s well-being. It is a consequence of various factors, including a compromised immune system resulting from a deteriorating intestinal environment. This, in turn, contributes to a decline in overall quality of life. In severe cases, constipation can lead to paralytic ileus, and if it progresses to intestinal ischemia or perforation, it can result in mortality.

A notable complication of severe constipation is Ogilvie’s syndrome, characterized by the sudden and severe expansion of the colon. This condition is characterized by a sudden and severe expansion of the colon in the absence of any physical obstruction [18]. The precise mechanism underlying this phenomenon still needs to be elucidated; however, one postulation suggests an imbalance within the autonomic nervous system, specifically between the sympathetic and parasympathetic nerves within the colon. This hypothesis suggests that a shift toward increased sympathetic nervous system activity, attributable to a diminution in the function of sacral parasympathetic nerves, results in a suppression of colonic peristalsis. The potential role of psychotropic drugs in disrupting this balance is a subject of ongoing research. In addition to antipsychotics, there have been reports of opioids, tricyclic antidepressants, clonidine, calcium channel blockers, and anti-Parkinson’s drugs [19].

**Figure 2:**
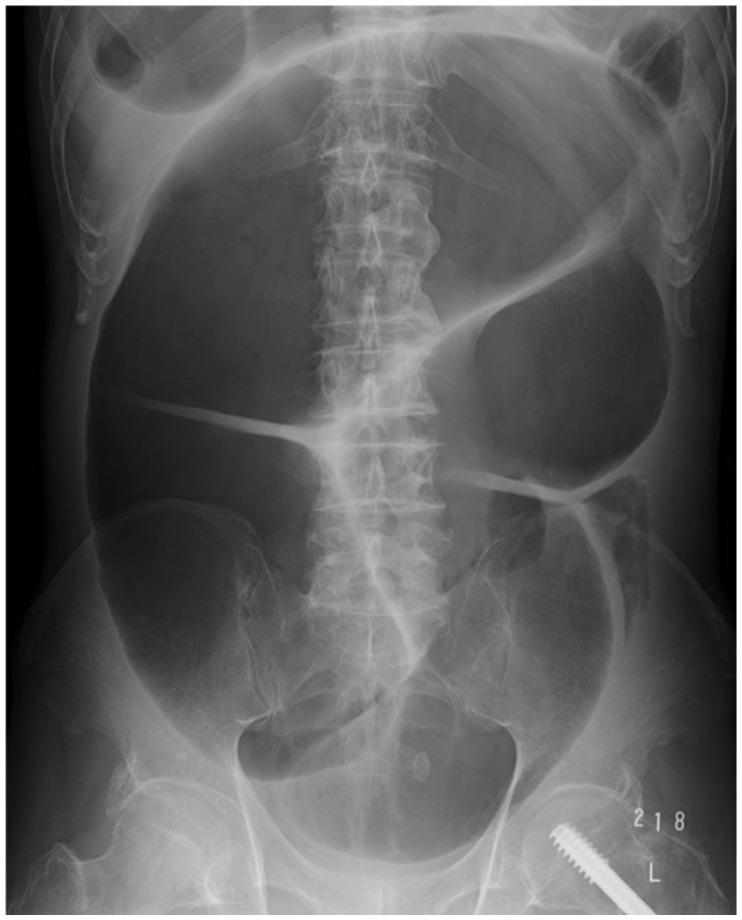
The photograph of this case shows the marked dilation of the large intestine that is typical of Ogilvie’s syndrome.

The diagnosis of this condition is not difficult when utilizing imaging techniques. A distended colon without a mechanical obstruction is visible on abdominal X-rays and CT scans. An illustrative abdominal X-ray from a case is presented in Figure 2. The case in question pertains to a 66-year-old female patient diagnosed with schizophrenia, who presented with acute abdominal distension. The patient had undergone osteosynthesis for a left femoral neck fracture several years prior and had been receiving 300 mg of clozapine for the treatment of schizophrenia. It is noteworthy that the patient had been experiencing chronic constipation. At the time of the onset of Ogilvie’s syndrome, the patient exhibited mild abdominal discomfort and was not vomiting. However, the patient subsequently developed sepsis due to the bacterial translocation caused by colonic distension. Blood cultures revealed the presence of Escherichia coli, leading to a further deterioration in the patient’s overall condition. The administration of psychotropic medications was suspended, and the patient was treated with decompression via a nasogastric tube and the infusion of meropenem. Following approximately 20 days of treatment, the patient demonstrated sufficient recovery to resume oral alimentation.

Clozapine has been reported most frequently among antipsychotics. In a case report of a 29-year-old male patient treated with clozapine, an abdominal CT scan revealed significant dilatation from the cecum to the ileum, with no evidence of gastrointestinal perforation. However, the patient subsequently developed symptoms of dyspnea, hypotension, and impaired consciousness, accompanied by primary metabolic acidosis with a normal anion gap and respiratory compensation. The patient ultimately succumbed to the complications 13 h after admission [20]. In a separate case of Ogilvie’s syndrome in a 60-year-old woman with a history of treatment-resistant schizophrenia (TRS) who was being treated with clozapine, she eventually developed massive bleeding and secondary intraperitoneal sepsis. Consequently, an emergency total colectomy and an ileostomy were performed to save her life [21]. A comprehensive report that encompassed 102 cases of life-threatening clozapine-induced intestinal dilatation revealed a mortality rate of 27.5%, with the majority of cases necessitating intestinal resection. Risk factors have been reported to include the initial start of clozapine administration, high doses of clozapine (high serum concentrations), and the concomitant use of anticholinergic drugs [22]. A conspicuous discrepancy exists between males and females with regard to constipation; however, Ogilvie’s syndrome, a condition precipitated by antipsychotics, manifests identically in both sexes [23]. This uncommon adverse effect may arise from the antipsychotics’ impact on domains other than the defecation reflex, such as muscle contraction, the autonomic nervous system in muscle tone, or the immune system [24].

**Figure 3:**
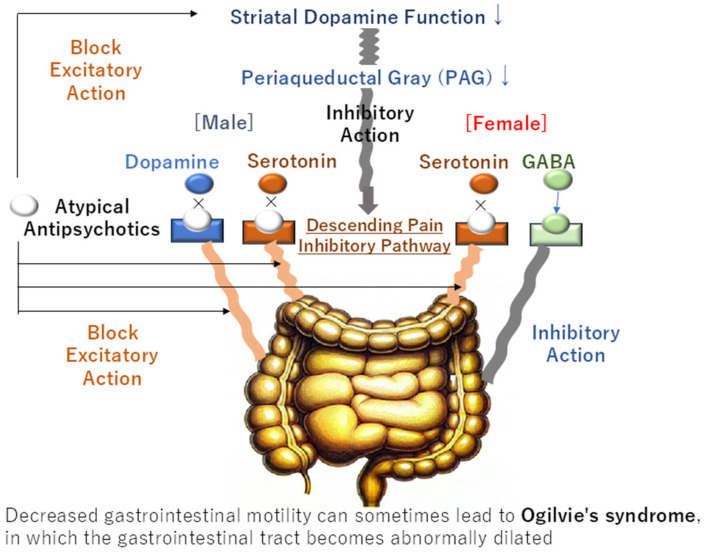
Explanation of the mechanism of atypical antipsychotic-induced constipation considering sex differences and its effect on causing serious complications.

Atypical antipsychotics have been shown to reduce the defecation reflex in the descending pain inhibitory system, which originates from the PAG. This effect is achieved by blocking dopamine in the central striatum. Additionally, these medications compete with dopamine and serotonin, which are excitatory neurotransmitters within the descending pain inhibitory system, further reducing the defecation reflex. In women, the defecation reflex is further reduced via GABA. This reduction in gastrointestinal motility can lead to gastrointestinal distension, a risk that is independent of sex. The underlying mechanisms and their implications are further elucidated in Figure 3.

### 4.5. Laxatives and Probiotics for Treating Constipation

In the treatment of constipation in patients taking antipsychotics, it is necessary to prescribe appropriate laxatives as well as adjusting the dose of the antipsychotic. However, there is a paucity of studies that directly compare the efficacy and adverse effects of various laxatives for constipation in individuals with schizophrenia, and guidance is lacking [25]. A search of the Cochrane Schizophrenia Group’s Trials Register also found that there was insufficient evidence to evaluate the efficacy and safety of pharmacological interventions for the treatment of antipsychotic-associated constipation due to limited and low-quality data [26].

The Japanese Society of Gastroenterology has published the initial edition of the 2023 Guidelines for the Treatment of constipation [27]. The initial step in the treatment of chronic constipation involves the modification of lifestyle habits and dietary therapy. The first pharmaceutical agent to be considered for oral treatment is an osmotic laxative. In cases where these prove ineffective, excretory agents and ileal bile acid transporter inhibitors are considered as further treatment options. The judicious use of stimulant laxatives is advised, with their application being reserved for specific cases. Probiotics, bulk laxatives, gastrointestinal motility enhancers, and herbal medicines are regarded as alternative or complementary therapies due to the absence of sufficient evidence, such as randomized controlled trials, and further research is necessary to substantiate their efficacy [27]. Thus, a multitude of digestive guidelines suggest that magnesium oxide is the optimal treatment for constipation. However, it has also been reported that magnesium oxide alters the pH of the intestine and the composition of the bacterial flora. In cases where the effect is insufficient, the administration of probiotics may prove effective [28]. In individuals diagnosed with schizophrenia who do not respond to laxatives, the administration of three probiotic strains has been shown to enhance the regularity and frequency of stools [29]. The efficacy of probiotics in ameliorating constipation in patients diagnosed with schizophrenia is a subject that remains the focus of ongoing research. It is acknowledged that individual variability may play a significant role in the response to probiotics. In a particular study, the administration of probiotics containing butyrate-producing bacteria led to a substantial alleviation of constipation in hospitalized patients diagnosed with schizophrenia, particularly after a treatment period of two months [29]. It is noteworthy that the efficacy of probiotics in ameliorating gut microbiota abnormalities may be more protracted than the immediate defecation effect of laxatives [30]. This outcome aligns with the prevailing notion that probiotics stimulate intestinal peristalsis by modulating the gut microbiota and systemic immune response. It has been posited that the decline in intestinal barrier function and the decline in butyrate-producing bacteria in individuals with schizophrenia are associated with psychiatric symptoms [31]. In animal models, probiotics have been demonstrated to restore not only bowel movements but also cognitive function [32]. Consequently, probiotics may be a viable consideration for schizophrenia patients experiencing constipation.

In patients receiving antipsychotics, constipation induced by these medications should be closely monitored. Timely intervention is necessary to prevent serious gastrointestinal effects. The efficacy of laxatives in this patient population remains a subject of debate, as evidenced by the absence of a consensus on their effectiveness [25].

### 4.6. Study Limitations

The most significant limitation of this study is the utilization of only two search engines. In subsequent research, it will be essential to comprehensively search papers by adding searches in the Web of Science, Embase, and the Cochrane Library, among others. Search terms should be expanded to include ileus, bowel disorders, decreased gastrointestinal motility, and unpleasant events associated with antipsychotics. The literature related to reduced QOL due to antipsychotic-related subjective adverse events should also be included. Moreover, research on constipation in patients with schizophrenia consists primarily of case reports and observational studies, which have been identified as being underestimated [33]. While Mendelian randomized trials have been conducted to examine the causal relationship between schizophrenia and constipation [34], the selection of genes may introduce variability in study outcomes. Consequently, randomized controlled trials are required to further investigate the association between schizophrenia and constipation.

## 5. Conclusions

The prevalence of constipation is high in individuals with schizophrenia, at approximately 30–60%. Moreover, there has been an observed increase in the prevalence of constipation in this patient population. The risk of constipation is increased by high doses of antipsychotics and by the concomitant use of anticholinergics and mood stabilizers. Atypical antipsychotics have been observed to elevate the risk of constipation, with clozapine demonstrating a particularly high association. Ogilvie’s syndrome, a grave complication of constipation, has been documented in numerous cases associated with clozapine. The current guidelines do not consider gender-related differences in the efficacy and tolerability of antipsychotics in patients with schizophrenia, yet the prevalence of constipation is higher in women than in men. Addressing this disparity necessitates the development of treatment regimens that consider gender-related differences. There is currently no consensus on the use of laxatives to treat constipation in patients with schizophrenia. The necessity of randomized controlled trials to investigate the efficacy of laxatives and probiotics for constipation in patients with schizophrenia is paramount.

## Data Availability

The data that support the findings of this study are available from the corresponding author upon reasonable request.
